# Complete Genome Sequence of a Mammarenavirus Harbored by Rodents on Hainan Island, China

**DOI:** 10.1128/genomeA.00129-18

**Published:** 2018-03-08

**Authors:** You Zhang, Langyu Rao, Gang Lv, Lei Zhang, Ruijia Fu, Shanshan Wang, Yue Wu, Xiuji Cui, Feifei Yin

**Affiliations:** a Hainan Medical University–The University of Hong Kong Joint Laboratory of Tropical Infectious Diseases, Hainan Medical University, Haikou, Hainan, China; b Key Laboratory of Translation Medicine Tropical Diseases, Hainan Medical University, Haikou, Hainan, China; c Department of Pathogen Biology, Hainan Medical University, Haikou, Hainan, China; d Core Facility and Technical Support, Wuhan Institute of Virology, Chinese Academy of Sciences, Wuhan, Hubei, China

## Abstract

Wenzhou virus (WENV) is a rodent-borne mammarenavirus that was recently found to infect humans. In this study, we sequenced and analyzed the complete genome of a genetic variant of WENV, HMU (Hainan Medical University) virus. The virus was harbored by a Rattus norvegicus individual in the residential areas of Hainan Province in southern China.

## GENOME ANNOUNCEMENT

Arenaviruses are enveloped single-stranded RNA viruses composed of two segments encoding four viral proteins (1, 2). The family Arenaviridae is classified into two genera, Mammarenavirus and Reptarenavirus. The genus Reptarenavirus represents arenaviruses from snakes, and at least 25 members of the genus Mammarenavirus whose primary natural reservoirs are rodents have been identified worldwide (3). The infected rodents can carry the viruses and transmit them to humans by close contact (4, 5). Mammarenaviruses cause central nervous system disease and hemorrhagic fever in people in both Africa and Latin America, and they contribute significantly to the human disease burden (6–8). So far, only three mammarenaviruses—the widely spread lymphocytic choriomeningitis virus (LCMV) and the recently described Wenzhou virus (WENV) and Loei River mammarenavirus (LORV)—have been identified in Asia (9–11). Both LCMV and WENV are found to infect humans, in whom they cause mild disease with symptoms similar to those of common viral infections. In this study, a genetic variant of WENV, HMU (Hainan Medical University) virus, was identified in a Rattus norvegicus individual captured in a residential area of Haikou City, Hainan Province, southern China. Viral RNA was extracted from anal swabs and subjected to high-throughput sequencing on a HiSeq 2000 platform (Illumina). Trinity version 2.0.6 was used for *de novo* assembly. Contigs were compared with sequences from the NCBI nucleotide database, and the WENV isolate Rn-242 (GenBank accession numbers KJ909795 and KJ909794) was identified as the most closely related genome to that of the HMU virus (10). Primers were designed to cover the gaps by PCR amplification and Sanger sequencing.

The HMU virus had two segments comprising 3,334 bp (small segment [S]) and 7,147 bp (large segment [L]), with G+C contents of 44.21% and 42.66%, respectively. Both S and L segments encoded two viral proteins in an opposite orientation. The S segment encoded an envelope glycoprotein (GP) of 492 amino acids (aa) and a nucleocapsid protein (NP) of 567 aa, and the L segment encoded an RNA-dependent RNA polymerase (RdRP) of 2,230 aa and a zinc-binding protein (ZP) of 91 aa. In both of the segments, the genes were separated by a noncoding region of 62 bp for the S segment and 109 bp for the L segment, which were proposed to play roles in transcription termination. Sequence analysis indicated that the genome of the HMU virus was most closely related to that of WENV isolate Rn-242, with identities of 94% and 95% for the L and S segments at nucleotide level, respectively. They were also closely related to each other at their structural proteins (GP, 97%; NP, 98%; RdRP, 96%; and ZP, 87%). Phylogenetic analysis showed that HMU virus was closely related to WENV and LORV and formed an independent clade with them within the genus Mammarenavirus (10, 11). Under the International Committee on Taxonomy of Viruses (ICTV) criteria for the classification of arenaviruses, HMU virus represents a genetic variant of WENV. The identification of HMU virus expands the geographic range of this mammarenavirus species and will inform future investigations of molecular pathogenesis of WENV in rodents and humans.

### Accession number(s).

The genome sequences of the S and L segments of the HMU virus have been deposited in GenBank under the accession numbers MF595889 and MF595888, respectively.
